# A Panel of Tumor-associated Autoantibodies for the Detection of Early-stage Breast Cancer

**DOI:** 10.7150/jca.57019

**Published:** 2021-03-05

**Authors:** Chao-Qun Hong, Xue-Fen Weng, Xu-Chun Huang, Ling-Yu Chu, Lai-Feng Wei, Yi-Wei Lin, Liu-Yi Chen, Can-Tong Liu, Yi-Wei Xu, Yu-Hui Peng

**Affiliations:** 1 Guangdong Provincial Key Laboratory of Breast Cancer Diagnosis and Treatment, Cancer Hospital of Shantou University Medical College, Shantou 515041, Guangdong, China.; 2 Department of Clinical Laboratory Medicine, Cancer Hospital of Shantou University Medical College, Shantou 515041, Guangdong, China.; 3 Precision Medicine Research Centre, Shantou University Medical College, Shantou 515041, Guangdong, China.; 4 Guangdong Esophageal Cancer Research Institute, Shantou University Medical College, Shantou 515041, Guangdong, China.

**Keywords:** breast cancer, early diagnosis, biomarker, autoantibody.

## Abstract

We previously found a panel of autoantibodies against multiple tumor-associated antigens (BMI-1, HSP70, MMP-7, NY-ESO-1, p53 and PRDX6) that might facilitate early detection of esophagogastric junction adenocarcinoma and esophageal squamous cell carcinoma. Here we aimed at assessing the diagnostic performance of these autoantibodies in breast cancer patients. Enzyme-linked immunosorbent assay was applied to detect sera autoantibodies in 123 breast cancer patients and 123 age-matched normal controls. We adopted logistic regression analysis to identify optimized autoantibody biomarkers for diagnosis and receiver-operating characteristics to analyze diagnostic efficiency. Five of six autoantibodies, BMI-1, HSP70, NY-ESO-1, p53 and PRDX6 demonstrated significantly elevated serum levels in breast cancer compared to normal controls. An optimized panel composed of autoantibodies to BMI-1, HSP70, NY-ESO-1 and p53 showed an area under the curve (AUC) of 0.819 (95% CI 0.766-0.873), 63.4% sensitivity and 90.2% specificity for diagnosing breast cancer. Moreover, this autoantibody panel could differentiate patients with early stage breast cancer from normal controls, with AUC of 0.805 (95% CI 0.743-0.886), 59.6% sensitivity and 90.2% specificity. Our findings indicated that the panel of autoantibodies to BMI-1, HSP70, NY-ESO-1 and p53 as serum biomarkers have the potential to help detect early stage breast cancer.

## Introduction

Breast cancer, the most commonly diagnosed cancer, has been the main leading cause of female cancer-related deaths worldwide 1. The incidence and mortality of breast cancer in female far exceeded those of other cancers 1. In China, females aged higher than 50 had higher risk of breast cancer, and at this age the death number accounted for more than 80% in all age groups 2. While the incidence of breast cancer has increased in recent decades, the death rate has steadily dropped owing to early detection and advancement in therapy 3. It is well known that breast cancer screening promotes the improvement of breast cancer prognosis. Despite the fact of mammography as a screening method to availably reduce the mortality of breast cancer, this technique is not routinely suited to detect tiny lesions. In fact, most breast cancer patients usually present at the late stage at diagnosis 4. For successful management of breast cancer, identification of specific noninvasive biomarker for early stage breast cancer is in demand. Although great efforts have been made in this research area, clinical applications are difficult to succeed and still in infancy 5. Noninvasive serum biomarkers are believed to be attractive tools in cancer diagnosis, as they have been found to predict risk of cancer and monitor molecular event in early variation of tumorigenesis 6.

Recently, autoantibodies against tumor-associated antigens (TAAs) are found to have enormous potential for the exploitation of circulating protein-based biomarkers and have been proposed as hopeful biomarkers for early breast cancer detection 7, 8. TAAs, which have altered protein expression levels, protein misfolding, or aberrant post-translational modifications, can elicit immune responses resulting in the production of autoantibodies 9-11. Importantly, autoantibodies are subjected to effective biological amplification, thus making them to be measured easily for detection compared to their corresponding TAAs in blood 11. Moreover, autoantibodies are particularly stable and less prone to degradation in blood sample, and can be detectable at early onset of cancer 12. Thus, these autoantibodies can be potentially developed as biosensors for recognizing cancer-related proteomic changes to develop valid diagnostic tools for early cancer detection. Autoantibodies in the prediagnostic sera are highly sought after in breast cancer 13-15. Though autoantibodies as informative markers in screening of early breast cancer are yet to emerge in clinical use, advance has been made to identify and validate promising autoantibody signatures in early diagnosis of breast cancer.

In our previous studies, we assessed the early diagnostic value of autoantibodies against a panel of six TAAs (BMI-1, HSP70, MMP-7, NY-ESO-1, p53 and PRDX6) in esophagogastric junction adenocarcinoma and esophageal squamous cell carcinoma, and the results were validated in independent cohorts 16, 17. Our results also indicated that single autoantibody detection showed poor diagnostic value but optimized panels of autoantibodies were more suitable to be used as an available tool for early cancer diagnosis. In this study, we explored the diagnostic performance of individual autoantibodies and aimed to identify an optimized autoantibody panel for detecting early-stage breast cancer.

## Materials and Methods

### Patient selection

Between 2013 and 2014, we collected sera samples of female breast cancer patients at the Cancer Hospital of Shantou University Medical College. This study was approved by the Ethical Review Board of Cancer Hospital of Shantou University Medical College, and informed consent was signed by all participants before sampling. Blood samples were processed in a similar way, which were collected at diagnosis before any treatments, centrifuged at 1,250*g* for 5 minutes, and stored at -80℃ until further use. All the breast cancer patients were confirmed by radiographic examination and histopathology, and cancer staging was done according to 8th edition AJCC. In this study, tumors with stages I + II + IIIA were defined as early stage disease according to the NCI Dictionary of Cancer Terms. Patients with other tumors or a history of tumors were excluded. And the samples of healthy control confirmed without evidence of malignancy by medical check-up were obtained during the same period.

### Enzyme‑linked immunosorbent assay (ELISA)

Two researchers (Chao-Qun Hong and Yi-Wei Xu) blind to clinical information performed the ELISA as previously described^16^, ^17^. Briefly, purified recombinant proteins of BMI-1, HSP70, MMP-7, NY-ESO-1, p53 and PRDX6, which were prepared in our previous works 17, were diluted in 50 mM bicarbonate buffer (pH 9.6) to 1.5, 0.8, 0.6, 0.1, 0.1, and 0.6 mg/mL, respectively. Quality control sample randomly collected from 50 breast cancer patients, and serum samples of subjects (diluted 1:110) were incubated at 37 °C for 1 h. After washing, horseradish peroxidase (HRP)-conjugated goat anti-rabbit IgG or anti-human IgG (Santa Cruz Biotechnology) as secondary antibodies were added for incubation. The plates were then washed. And were added for color formation. Finally, we used a plate microplate reader to read the absorbance at 450 nm/630 nm of each well.

### Statistical analyses

SPSS or GraphPad Prism software were used for statistical processing. Mann-Whitney's U test was used to compare the significant differences in autoantibodies levels between cancer and control subjects, and the Chi-squared test was applied to assess the relationship between clinical features and autoantibody positive rate. Receiver operating characteristic (ROC) analysis was performed to evaluate the diagnostic performance and to obtain the cut-off according to the criterion described in previous study 17. The specificity over 90% is believed to make a diagnosis test to be beneficial to early detection of cancer 18. To select an optimized panel of autoantibody biomarkers for diagnosis, we applied logistic regression analysis, and then constructed ROC curve by using the predicted probability of being diagnosed with breast cancer as one marker. Differences were considered statistically significant when *P* value < 0.05.

## Results

### Participant characteristics

**Figure 1:**
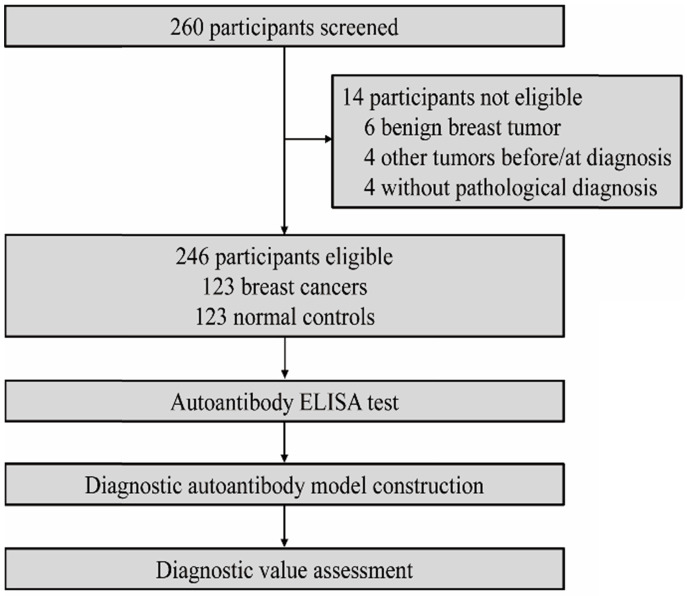
Study profile.

We recruited 246 participants overall, with 123 in patient group and 123 in control group (Figure 1). The two groups were age-matched. Patient details and tumor characteristics are summarized in the Table 1. In this study, there were 94 patients identified as early-stage breast tumor diseases.

### Autoantibody levels in breast cancer

**Figure 2:**
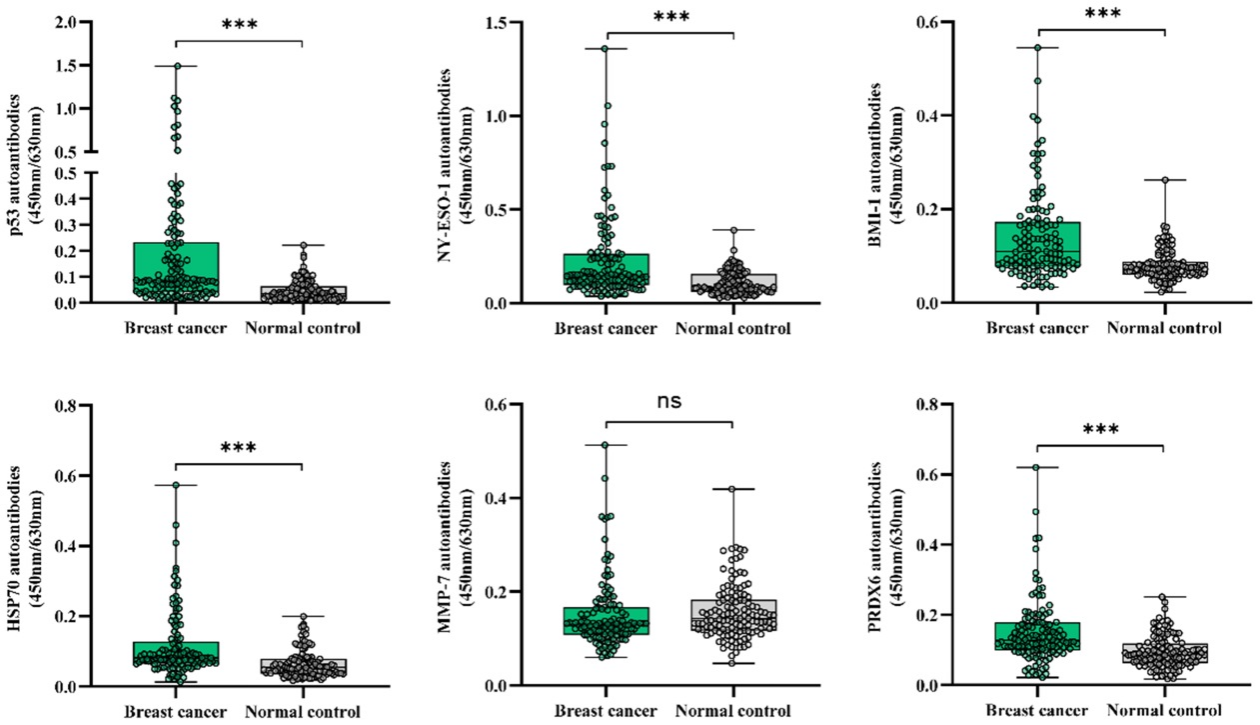
Scatter plot and box plot for serum levels of individual autoantibodies in breast cancer. ^***^P < 0.001, ns, no significance.

Figure 2 shows serum levels of individual autoantibodies in the detection of breast cancer disease group and normal control group. Mann-Whitney's U test exhibited that levels of serum autoantibodies to BMI-1, HSP70, NY-ESO-1, p53 and PRDX6 were significantly higher in breast cancer patients than those in controls. As levels of MMP-7 autoantibodies were not elevated in patients with breast cancer, we excluded it for further analysis in this study.

### Diagnostic value of autoantibodies in breast cancer

**Figure 3:**
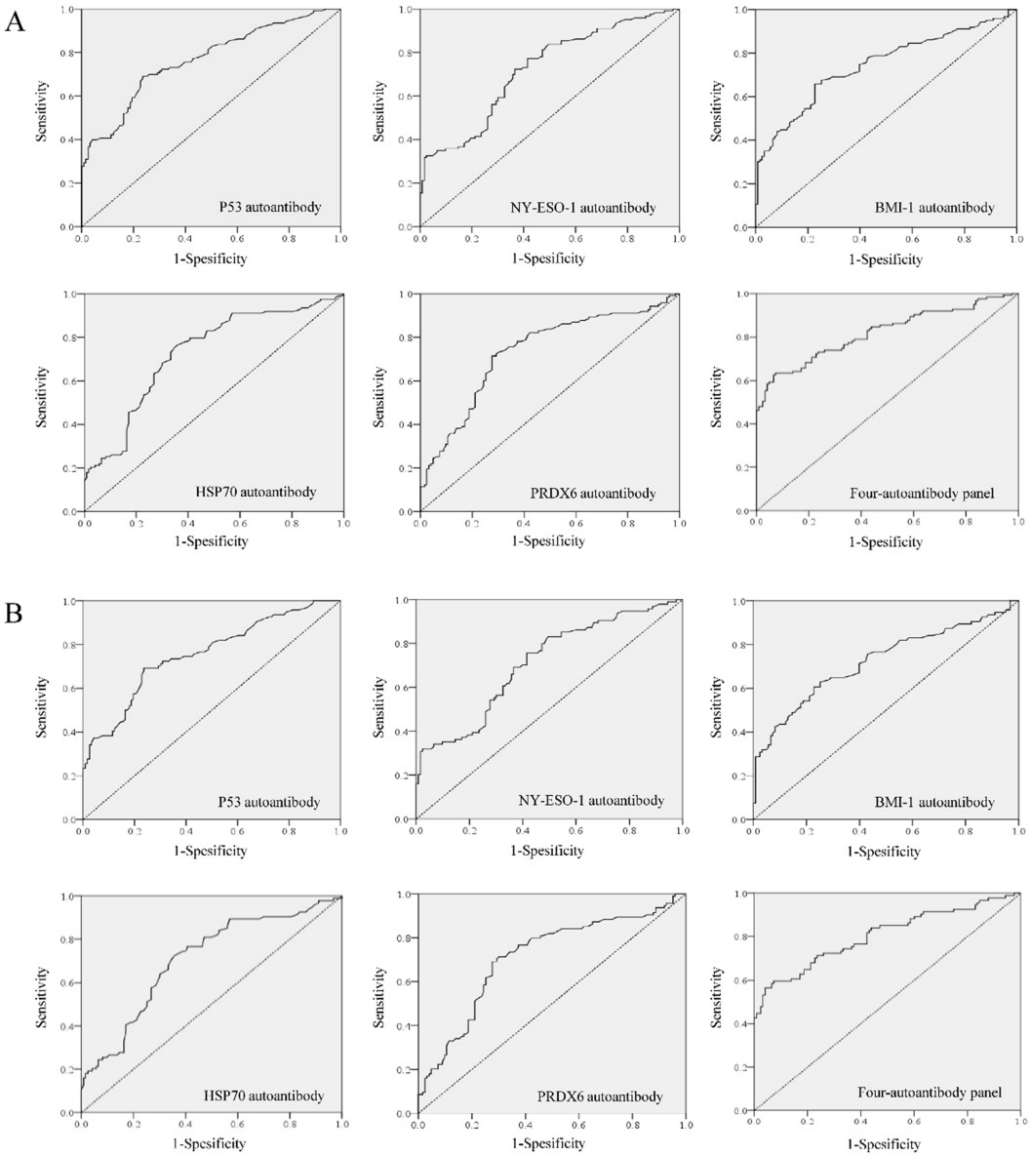
Diagnostic performance of autoantibodies to diagnose breast cancer. A. ROC curve analysis for individual autoantibodies and the panel comprising BMI-1, HSP70, NY-ESO-1 and p53 autoantibodies for all patients with breast cancer vs. controls. B. ROC curve analysis for individual autoantibodies and the panel comprising BMI-1, HSP70, NY-ESO-1 and p53 autoantibodies for patients with early stage breast cancer vs. controls.

ROC curves demonstrated the optimum diagnostic cutoff OD values for serum autoantibodies against BMI-1, HSP70, NY-ESO-1, p53 and PRDX6 were 0.126, 0.123, 0.193, 0.105 and 0.161, respectively. As shown in Table 2, compared with normal controls, the positive percentages in individual autoantibody detections were all increased in both breast cancer and early-stage breast cancer patients (*P*<0.01). In order to identify an optimize autoantibody panel, we applied a forward stepwise logistic regression analysis to score the predicted probability (p) of being diagnosed with breast cancer based on the autoantibody dataset from 246 samples from all cancer patients and normal controls. As a result, autoantibodies against BMI-1, HSP70, NY-ESO-1 and p53 were identified to be valid predictors, with the p value calculated by ln[p/(1 - p)] = 8.628 × (BMI-1) + 6.960 × (HSP70) + 5.166 × ( NY- ESO -1) + 11.724 × (p53) - 3.088. Then the p was used to establish the ROC curve (Figure 3). When the cut-off was set at 0.542, the AUC for this optimized autoantibody panel were 0.819 (95% CI, 0.766 to 0.873), with the sensitivity of 63.4% and the specificity of 90.2% (Table 3). It was obvious that the diagnostic performance of the autoantibody panel for breast cancer was improved when compared to individual autoantibodies, of which the AUCs and sensitivities ranged from 0.721 (NY-ESO-1 autoantibody) to 0.766 (p53 autoantibody), and from 25.2% (HSP70 autoantibody) to 40.7% (p53 autoantibody), respectively. Importantly, we found almost the same diagnostic efficiency in the early-stage breast cancer patients (AUC 0.805, sensitivity 59.6% and specificity 90.2%) by using the same cutoff for the autoantibody panel, of which the diagnostic value was also improved when compared with individual autoantibodies (Figure 3 and Table 3).

### Autoantibody and clinicopathological parameters of breast cancer

**Figure 4:**
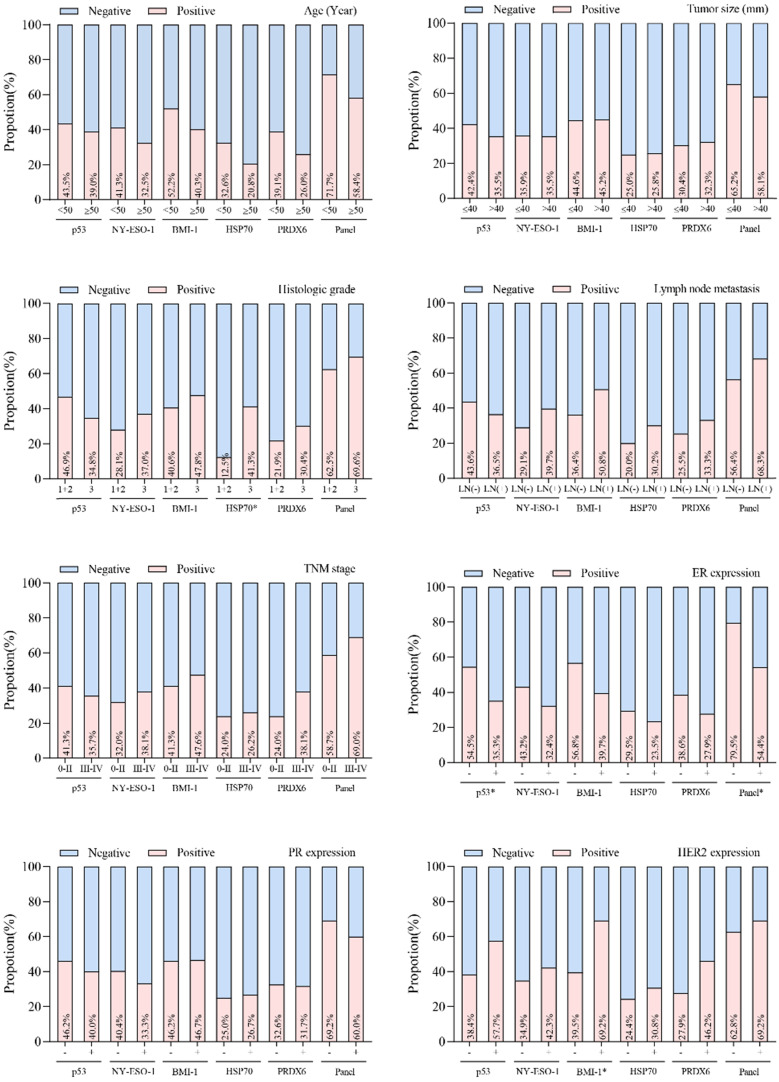
Relationship between positive rates of autoantibodies and the clinicopathological variables in breast cancer patients. ^*^P < 0.05.

The association between individual autoantibodies or the autoantibody panel and clinical characteristics was assessed for breast cancer cases. No correlation of autoantibody properties to tumor size, lymph node status, TNM stage, age or PR expression was seen in breast cancer patients (Figure 4). Positive rates of autoantibodies against HSP70, p53 and BMI-1 were observed to be influenced by histologic grade, ER expression and HER2 expression states, respectively. In addition, the autoantibody panel also demonstrated a significant correlation to ER expression (*P*<0.05).

## Discussion

Tumor-associated autoantibodies have become one of the most important areas in biomarker exploitation for early cancer detection, as autoantibody detection could precede the clinical symptoms of breast cancer 7, 13, 19. Increasing studies have shown valuable role of autoantibodies in the diagnosis, prognosis and prediction of treatment effect of many types of cancers 20, 21. Our study demonstrated that individual autoantibodies can be detected in a range of 25.5-43.6% of patients with early breast cancer, and the sensitivity was improved to 59.6% when autoantibodies against BMI-1, HSP70, NY-ESO-1 and p53 built as an optimized panel were measured simultaneously. Thus, combined detection of serum autoantibodies could improve results.

Mammography for screening of breast cancer has been commonly used in many high-income countries. But this tool also has limitations that have been a globally concerned issue for decades 22-25. Among these, overdiagnosis and radiation are the main concerns for mammography. Breast cancer overdiagnosis by mammogram has been considered as a significant adverse event, as the overdiagnosis probabilities were estimated ranging between 0% and 40-50% depending on subject age and approaches 8, 26, 27. Moreover, this technique is not suited for detecting small-size tumors, especially in women with high breast density 28, 29. It is consequently imperative to develop complementary tools for early detection. In recent years, great efforts have been made on the development of blood-based biomarkers which show the potential for earlier detection of breast cancer. Serum biomarkers CEA and CA15-3 are routinely used in clinical settings for breast cancer but they show insufficient sensitivity and specificity. What is encouraging is that autoantibodies are now developing as promising biomarkers for detecting cancers at an early stage. The EarlyCDT-Lung test that measures seven cancer autoantibodies has been proved to contribute to predicting lung cancer risk, and has been clinically used in the risk evaluation for malignancy in vague pulmonary nodules 30, 31. For breast cancer, numerous serum autoantibodies have been reported with potential early diagnostic value, whereas only few have been investigated in detail to evaluate the diagnostic utility. To date, individual autoantibodies are shown to have low clinical sensitivity, thus cannot be applied to screen early breast cancer 7. To resolve the issue of low sensitivity for early diagnosis, increasing studies have developed autoantibody panels 32-34. For example, Chapman et al. measured multiple autoantibodies against tumor-associated antigens (BRCA1, BRCA2, c-myc, HER2, MUC1, NY-ESO-1, and p53), and observed that sensitivities for individual autoantibodies varied between 8-34% and 3-23% in primary breast cancer and ductal carcinoma *in situ* patients, respectively. While 45% of ductal carcinoma *in situ* and 64% of primary breast cancer patients showed positive results of the combined autoantibody panel at 85% specificity 35. In this study, we also gave evidence to boost encouraging results of the measurement of autoantibody panels. Nevertheless, these markers for breast cancer screening are at early development phase, and miscellaneous issues in preclinical and clinical settings are still required to be addressed.

In our previous studies, we found that serum levels of autoantibodies against BMI-1, HSP70, MMP-7, NY-ESO-1, p53 and PRDX6 were all significantly elevated in sera of esophageal squamous cell carcinoma patients and gastroesophageal junction adenocarcinoma patients, compared to controls 16, 17. Here we identified that no significant difference was observed in the serum level of MMP-7 autoantibodies between breast cancer patients and normal, indicating that this marker might not be used as a diagnostic biomarker in breast cancer. However, this result should be further validated by using large samples. On the other hand, we noted that the diagnostic efficiency of the autoantibody panel comprising BMI-1, HSP70, NY-ESO-1 and p53 autoantibodies for early stage breast cancer was similar with other optimized autoantibody panels identified for early stage esophagogastric junction adenocarcinoma and esophageal squamous cell carcinoma 16, 17. It is apparent that the efficacy of individual autoantibodies in the panel varied in different cancer types, suggesting the heterogeneity of cancer and the importance of proper combination in a certain cancer type. What we need to do in the next stage of work should be focus on the identification and selection of specific autoantibodies for breast cancer. In addition, according to the recommended five-phase schema and the Prospective sample Collection Retrospective Evaluation (PRoBE) guidelines for biomarker evaluation study^36^, ^37^, our current study belongs to Phase 1/2 in biomarker development, thus needing to conduct blinded validation studies and retrospective longitudinal studies (Phase 3) to detect preclinical disease. What's more, mammography is proved to assist in early detection of breast cancer in clinical practice^38^. It would better to display the diagnostic accuracy comparison between our autoantibody panel and mammography, or to evaluate whether the combination of them could improve the early diagnostic efficiency.

In summary, our study presented an autoantibody panel which might be proposed as an easy and noninvasive tool to help identify early-stage breast cancer. Considering the limitations of single center study, small sample size and the lack of assessment of the combined detection of mammography and our autoantibody assay in the present work, we need to carry out more investigation and validation to estimate the potential clinical value of this combined autoantibody assay.

## Figures and Tables

**Table 1:** Clinicopathologic characteristics of breast carcinomas

Characteristics	Primary breast cancer	Normal control
Mean Age ± SD (years)	53 ± 9	53 ± 11
Age range (years)	29 - 79	40 - 87
Tumor size range (mm)	7 - 120	
Size groupings		
<20 mm	15	
20-40 mm	77	
> 40 mm	31	
Histology		
Ductal	114	
Lobular	6	
Other	3	
Histologic grade		
1	5	
2	27	
3	46	
Missing	45	
TNM stage		
Ⅰ	19	
Ⅱ	56	
Ⅲ	39	
Ⅳ	3	
Missing	6	
Lymph node status (positive)	63	
ER		
Negative	44	
Positive	68	
Missing	11	
PR		
Negative	52	
Positive	60	
Missing	11	
Her-2		
Negative	86	
Positive	26	
Missing	11	

**Table 2:** Frequency of autoantibodies to tumor-associated antigens and the autoantibody panel

Group	p53	NY-ESO-1	BMI-1	HSP70	PRDX6	Panel
All breast cancer (n=123)	40.7%^**^	35.8%^**^	44.7%^**^	25.2%^*^	30.9%^**^	63.4%^**^
Early-stage breast cancer (n=94)	38.3%^**^	35.1%^**^	43.6%^**^	25.5%^*^	26.6%^*^	59.6%^**^
Normal controls (n=123)	9.8%	9.8%	9.8%	9.8%	9.8%	9.8%

Panel: autoantibody positivity to any one of the four antigens (p53, NY-ESO-1, BMI-1and HSP70). *P* value is relative to normal controls (χ ^2^ tests). ^*^P < 0.01; ^**^P < 0.001.

**Table 3:** Diagnostic results for the individual autoantibodies and the autoantibody panel in breast cancer

	AUC (95%CI)	Sensitivity	Specificity	PPV	NPV	PLR	NLR
**All stages**							
p53 autoantibody	0.766 (0.708-0.825)	40.7%	90.2%	80.6%	60.3%	4.15	0.66
NY-ESO-1 autoantibody	0.721 (0.658-0.784)	35.8%	90.2%	78.5%	58.4%	3.65	0.71
BMI-1 autoantibody	0.743 (0.681-0.805)	44.7%	90.2%	82.0%	62.0%	4.56	0.61
HSP70 autoantibody	0.728 (0.665-0.792)	25.2%	90.2%	72.0%	54.7%	2.57	0.83
PRDX6 autoantibody	0.734 (0.671-0.797)	30.9%	90.2%	75.9%	56.6%	3.15	0.77
Four- autoantibody panel	0.819 (0.766-0.873)	63.4%	90.2%	86.8%	71.1%	6.47	0.41
**Early stage**							
p53 autoantibody	0.756 (0.691-0.821)	38.3%	90.2%	74.9%	65.7%	3.91	0.68
NY-ESO-1 autoantibody	0.710 (0.641-0.779)	35.1%	90.2%	73.2%	64.5%	3.58	0.72
BMI-1 autoantibody	0.723 (0.652-0.793)	43.6%	90.2%	77.3%	67.7%	4.45	0.63
HSP70 autoantibody	0.707 (0.637-0.777)	25.5%	90.2%	66.5%	61.3%	2.60	0.83
PRDX6 autoantibody	0.711 (0.640-0.782)	26.6%	90.2%	67.5%	61.7%	2.71	0.81
Four- autoantibody panel	0.805 (0.743-0.866)	59.6%	90.2%	82.3%	74.5%	6.08	0.45

CI, exact confidence interval; NLR, negative likelihood ratio; NPV, negative predictive value; PLR, positive likelihood ratio; PPV, positive predictive value. Four-autoantibody panel: autoantibodies against p53, NY-ESO-1, BMI-1 and HSP70.

## Abbreviations

TAAs: tumor-associated antigens
BMI-1: BMI1 polycomb ring finger oncogene
HSP70: heat shock protein 70
MMP-7: matrix metalloproteinase-7
PRDX6: peroxiredoxin VI
ELISA: enzyme‑linked immunosorbent assay
HRP: horseradish peroxidase
ROC: receiver operating characteristic
AUC: area under ROC curve
OD: optical density
CI: exact confidence interval
NLR: negative likelihood ratio
NPV: negative predictive value
PLR: positive likelihood ratio
PPV: positive predictive value
PR: progesterone receptor
ER: estrogen receptor
HER2: human epidermal growth factor receptor-2
CEA: carcinoembryonic antigen
CA15-3: carbohydrate antigen 15-3
BRCA1: breast-cancer susceptibility gene 1
BRCA2: breast-cancer susceptibility gene 2
MUC1: Mucin 1
